# Clinical Remarks on Acute Articular Rheumatism

**Published:** 1861-03

**Authors:** Francis G. Smith

**Affiliations:** Attending Physician


*Clinical Remarks on Acute Articular Rheumatism at the Pennsylvania Hospital, February* 13, 1861. By Francis G. Smith, M. D., *Attending Physician.*

John Eulen, a native of Belgium, two years and a half a resident of this country, has been sick for seventeen days with acute articular rheumatism.

In presenting the case to the class Dr. Smith made the following remarks: When I took charge of the wards, on the first of the month, I found this man sick in bed with acute articular rheumatism. He was attacked about a week before I came on duty with fever, hot skin, profuse perspiration, pain and heat in the joints, but had no diarrhœa. He presented, in other words, well-marked symptoms of rheumatic fever, with articular implication. There was presented that peculiar, flushed look often seen in acute articular rheumatism; the surface was perspiring, emitting the peculiar acid smell; the pulse was full and tense; he had pain on moving the joints, which is characteristic of this disease. As you see him now these symptoms have disappeared entirely, so that he can walk without pain, sleep without being disturbed, and considers himself well. This is a disease constantly met with. Perhaps no disease falls more frequently under the observation of the physician than acute rheumatism, whether it presents itself in the form of rheumatic fever, or whether complicated with articular affections. It will not be out of place, therefore, to express some views in regard to its pathology, and also in regard to what has been a successful plan of treatment.

In the first place, what is the pathology of this disease, and upon what does it depend?

Is it purely a local disease manifested in inflammation of certain joints, or does it depend on the existence of a poison in the blood?

Gout, we know, depends on the existence in the blood of lithic acid, which results from the retrograde metamorphosis of the products of animal food introduced into the system over and above its wants. Those who indulge in the pleasures of the table, eat largely of animal food, and take but little exercise, take into their body more nutritive material than is necessary to make up for the wear and tear continuously going on in the system. If a man eats a large quantity of animal food, and takes sufficient exercise to burn up all the excess, he will probably escape disease; but, if in connection with luxurious living, he leads an indolent life, and takes an insufficient amount of oxygen into his system, that food will undergo changes in its backward progress into creatine, creatinine, uric acid, urea.

These changes require the introduction of oxygen into the blood,—in other words, a certain amount of combustion must take place for their production; if, therefore, a man takes exercise sufficient to carry on all these retrograde metamorphoses, the surplus matter will make its escape from the system in the form of urea. If it stop short of this, the uric acid will accumulate in the blood, and is regarded as the materies morbi of gout.

Rheumatism depends on the existence in the blood of an analogous poison—lactid acid—in consequence of a defect in the primary or secondary assimilation of the food as taken into the system. One reason why it prevails more than gout among the poor, is that the food which they consume is more liable to be converted into lactic acid than that consumed by the better classes of society. In the higher classes, where animal food is mostly indulged in, lithic acid is the result of the metamorphosis, while in the poorer classes, who rely on farinacious food, the transformation of the latter is into lactic acid.

Whenever this material accumulates in blood in large quantities, in consequence of an inability to undergo the transformation into carbonic acid, every effort is made on the part of nature to eliminate it from the whole system. We thus find it in the acid perspiration met with in rheumatism. I have noticed the effect of this acid perspiration on litimus paper, by putting it in contact with the bedewed surface of the skin, and seeing it change from blue to red.

It is, however, frequently observed that the profuse sweating which is so constantly seen in this disease, does not always relieve it, and it seems, therefore that this outlet is not sufficient for the elimination of the poison. There are other outlets by which it attempts to escape from the system; among these are, especially, those surfaces which we find well supplied with blood, and where the blood vessels are comparatively naked; where the blood circulating in them is but little expoosed to any detention from the glandular structures, and where, therefore, their contents are most readily able to escape. Thus, we find the serous membranes, the peritoneum, pleura, pericardium, even the arachnoid membrane, but more especially the synovial membranes of the joints, to be some of the outlets from which this materies morbi endeavors to escape. In a patient affected with acute articular rheumatism, the synovial membranes are the localities where the disease most frequently manifests itself; congestion takes place in them; there is a large supply of blood, in consequence of which, a diseased synovia is poured out, loaded with this acid secretion, and this, acting as an irritant, produces pain in the joint, and causes that swelling and bulging of the synovial capsules so constantly seen in the disease. This is the reason why you find in the large joints, heat, swelling and redness,

The synovial capsules are not the only ones liable to be affected; it is not unusual to find a patient with rheumatic fever laboring under peritoneal rheumatism, or even peritoneal inflammation—an accident not very common, it is true, but which does occur sometimes. Pleuritis is not an unusual complication, while pericarditis is so common and frequent, that many pathologists contend that you never meet a case of acute articular rheumatism, without having, at the same time, a pericardial inflammtion.

Endocarditis is also a common complication; in fact, in all these surfaces where the blood escapes with much difficulty we may have manifestations of disease presenting themselves; sometimes even we have pneumonia.

There are other materials capable of producing these same symptoms. There is a puerperal articular rheumatism from a materies morbi derived from the internal surface of the uterus. The matters detained contaminate the whole blood, and often give rise to a form of rheumatic fever, accompanied with swelling in the joints, sometimes very painful, going further than in ordinary acute articular rheumatism, so as to produce inflammation, ulceration, destruction of the articular surfaces, and filling the joints with pus. From the simple introduction of certain noxious merterials in the blood, the whole train of symptoms follow; they act as a ferment, and nature endeavors to eliminate them in the manner described.

If this be the theory of rheumatism—and there seem to be strong reasons for accepting it, as ably illustrated in the late Dr. Todd's clinical lectures— what should be the proper plan of treament?

What is the plan of treatment in all cases where there is a poison in the blood? Certainly, elimination.

In scarlantina the endeavor of nature, and the object of treatment, is to eliminate the poison; so in small-pox, and indeed in all exanthemata, depending on the presence of a poison in the blood.

In typhus and typhoid fever where there is an introduction of a poison in the blood, certainly the safest plan of treatment is elimination with sustentation.

In articular rheumatism we see that the skin is endeavoring to throw off this acid by profuse perspiration. Let us then trust her, and help nature, and try to assist in throwing off the poison by the administration of diaphoretics. This we can readily do by a class of diaphoretics, the salines, which are known to have a direct tendency toward the cutaneous surface. Another symptom of the disease, which it is important to meet, is the pain. Two indications, therefore, present themselves—first, to assist the elimination, and, secondly, to relieve pain.

By combining the saline diaphoretic with some opiate and a little ipecacuanha, we have fulfilled all these indications.

If the pain is not very severe we may omit the opium altogether, and adopt a more thoroughly eliminating process by the administration of some of the carbonates of the alkalies.

In the case presented to the class, the plan pursued was to give three times daily ten grains of the acetate of potash, (the old sal diureticus,) in combination with an equal quantity of carbonate of potassa, so that the patient should take three times a day twenty grains of the mixture. It was given freely in diluent drinks. It produced free diuresis, and, besides, caused an unusual amount of fluid to be eliminated in the course of twenty-four hours by diaphoresis also. Through these two instrumentalities the poison seems to have been thoroughly eliminated from the system. While doing this, we were careful to sustain the patient's strength; for in all cases where blood-poisoning exists, there is a tendency on the part of the poison to depress the powers of the system, and to produce a toxical influence on the nervous system, which manifests itself in delirium, or extreme depression of the nervous powers. We give, therefore, animal broths, with a slight amount of stimulus. We alse endeavored to eliminate this poison from his blood by local applications, tending to increase the sweating powers of his joints; they were closely wrapped up in carded wool, carefully prepared; as this is compressible into all forms and shapes, it could be wrapped all around the limb or painful joint. Outside of this, an envelope of oiled silk was placed, which kept up a profuse sweating, so that when removed, after having been on for twenty-four hours, the silk was completely loaded with perspiration.——*Med. & Surg. Reporter.*

